# Efficacy of 5-Fluorouracil in the Treatment of Pterygium

**DOI:** 10.7759/cureus.12652

**Published:** 2021-01-12

**Authors:** Sobia U Shah, Tanveer Ahmed, Anum Badar, Maeirah Shafique, Sidra Malik, Bushra Aaqil

**Affiliations:** 1 Ophthalmology, Combined Military Hospital (CMH) Lahore Medical College, National University of Medical Sciences (NUMS), Lahore, PAK; 2 Ophthalmology, Combined Military Hospital (CMH) Lahore, Lahore, PAK; 3 Ophthalmology, Combined Military Hospital (CMH) Rawalakot, Rawalakot, PAK; 4 Ophthalmology, Combined Military Hospital (CMH) Abbottabad, Abbottabad, PAK; 5 Ophthalmology, Pakistan Air Force (PAF) Faisal Base Karachi, Karachi, PAK; 6 Ophthalmology, Ayub Medical College, Abbottabad, PAK

**Keywords:** pterygium, 5 fluorouracil, efficacy, primary pterygium, recurrent pterygium, 5fu

## Abstract

Objective

To determine the efficacy of 5-Fluorouracil (FU) in the treatment of pterygium.

Methodology

After meeting the inclusion criteria 101 patients were enrolled in this study. Informed consent and demographic information was taken from all the patients. Patients underwent ophthalmic clinical examination that included slit lamp examination to grade pterygium. Before starting 5-FU injections, all topical medication was stopped. After four weeks the effects of 5-FU and its efficacy was noted. The patients were reviewed again after six months to note any recurrence. All the collected data was entered and analyzed on Statistical Package for Social Sciences (SPSS) version 20 (IBM Corp., Armonk, NY).

Results

In our study the mean age of the patients was 37.74 ± 10.15 years, male to female ratio of the patients was 1.06:1. The primary type of pterygium was noted in 54 (53.5%) and recurrent was noted in 47 (46.5%) patients. The efficacy achieved in 88 (87.13%) patients, four had recurrence of pterygium and of 101 patients 26 underwent surgical excision.

Conclusion

The use of 5-FU is safe and effective for the treatment of pterygium and it can be implemented as a primary treatment especially in the hot temperate zone where it is very common and aggressive with high recurrence rate. 5-FU not only halts its progression but also reduces the size and vascularity thus decreasing the need for surgery and steroid use and preventing recurrence.

## Introduction

Pterygium is a triangular growth of fibrovascular conjunctival tissue that typically starts medially on the nasal conjunctiva and extends laterally on to the cornea. It refers to shape of the tissue which looks like an insect wing [1]. Pterygium is unlikely to threaten visual acuity unless it approaches cornea nevertheless it can be a cause of concern to the patients because of abnormal appearance it confers upon the eye and the irritation that is often associated with it [2]. Prevalence of pterygium varies from 0.7% to 33%, being more common in males. It is associated with chronic sun exposure and specifically to ultraviolet light, thus it is more commonly seen in outdoor workers and in tropic and equatorial zone [3]. The standard treatment is excision with autologus conjunctival graft to cover the defect [4]. Recurrence is the most common complication after pterygium surgery which rates from 10 to 80% depending on excision procedure [3-5]. 5-Fluorouracil (5-FU), an anti-metabolite, is a pyrimidine analogue that causes apoptosis of fibroblasts by interfering with DNA and RNA synthesis of fibroblasts in the tenons capsule. In ophthalmology it is being used widely due to its anti-scarring properties in glaucoma filtering surgery and also in cases of recurrent pterygium [6,7]. Studies show that 93.3% patients had regression of fibrovascular tissue (thickness and vascularity) and arrest of progression following a dose of 0.1-0.2 ml of 5-FU in recurrent pterygium [1,8].

The rationale of this study is to see the efficacy of 5-FU on pterygium in our population and to determine whether or not 5-FU should be made a part of primary treatment of pterygium. Work has been done to see the effect of 5-FU in halting pterygium recurrence but very little work has been done where it is being assessed as a primary treatment modality thus decreasing the excessive use of steroids before and after surgery, preventing recurrence and need of surgical excision.

## Materials and methods

Patients reporting to outpatient department with pterygium of 2 or more millimeter over cornea with a grade of 2 or 3 were included in the study. Grade was assigned according to the following criteria: GRADE 1 (Atrophic pterygium) (Episcleral vessels clearly seen), GRADE 2 (Intermediate pterygium) (Episcleral vessels not clearly seen), GRADE 3 (Fleshy pterygium) (Episcleral vessels obscured). Patients with recent history of ocular trauma, corneal degeneration, scarring or ectasia were excluded. History was taken and age, gender, occupation, recurrence or any previous treatment modalities were documented. Patients underwent ophthalmic clinical examination that included slit lamp examination to grade pterygium. Case note entries were used to assess improvement after injection in both primary and recurrent pterygium. Approval was taken from ethical committee and informed written consent was taken from all individuals. Before starting 5-FU injections, all topical medications were stopped. 0.1 ml of 5-FU (5 mg) in 1 ml syringe was given intralesional under topical anesthesia. One to two drops of 5% povidone iodine was instilled in conjunctival sac 5 min before injection that was given in outpatient clinic using slit lamp. After injection Moxifloxacin was instilled and given Q.I.D for three days. Total of three injections were given at weekly intervals with follow-up after four weeks and effects of 5-FU noted and efficacy was determined in terms of improvement in the grade of pterygium ≥ 1 grade on slit lamp examination on follow-up visits after four weeks of last injection. The patients were seen after six months to record any recurrence in terms of a grade increase of >1. All the data was recorded on a specially designed proforma. Data was entered and analyzed by using Statistical Package for Social Sciences (SPSS) version 20 (IBM Corp., Armonk, NY). Pre- and post-injection clinical appearance and grading of pterygium was compared by chi-square test and frequency distribution was used to determine efficacy. Effect modifiers like age, steroid use and other ocular pathology were controlled through stratification.

## Results

The mean age of the patients was 37.74 ± 10.15 years with minimum and maximum ages of 21 and 55 years, respectively. There were 52 (51.49%) males and 49 (48.51%) females. The male to female ratio of the patients was 1.06:1. The study results showed that the primary type of pterygium was noted in 54 (53.5%) and recurrent was noted in 47 (46.5%) patients (Table 1).

**Table 1:** Frequency distribution of type of pterygium

		Frequency	Percent
Type	Primary	54	53.5
	Recurrent	47	46.5
	Total	101	100.0

The intermediate grade at baseline was noted in 46 (45.5%) patients and fleshy grade at baseline was noted in 55 (54.5%) patients. After 4th weeks it was found that 88 (87.1%) patients had improvement in their grade at baseline by ≥1 and 13 (12.9%) patients did not have any improvement in the grade at baseline a month after three injections of 5-FU. Post treatment cases showing higher number in grade 2 as out of 101 patients in the study 55 had a grade 3 pterygium which improved to grade 2 or 1 (Table 2).

**Table 2:** Frequency distribution of various grades of pterygium before and after treatment.

Clinical Appearance	Primary Pterygium		Recurrent Pterygium			P < 0.001 significant
	Pre-Treatment	Post-Treatment	Pre-Treatment	Post-Treatment		
Grade 1	0	27	0	15		P < 0.001
Grade 2	29	23	17	27		P < 0.001
Grade 3	25	4	30		5	P < 0.001
Total	54		47			101

**Figure 1:**
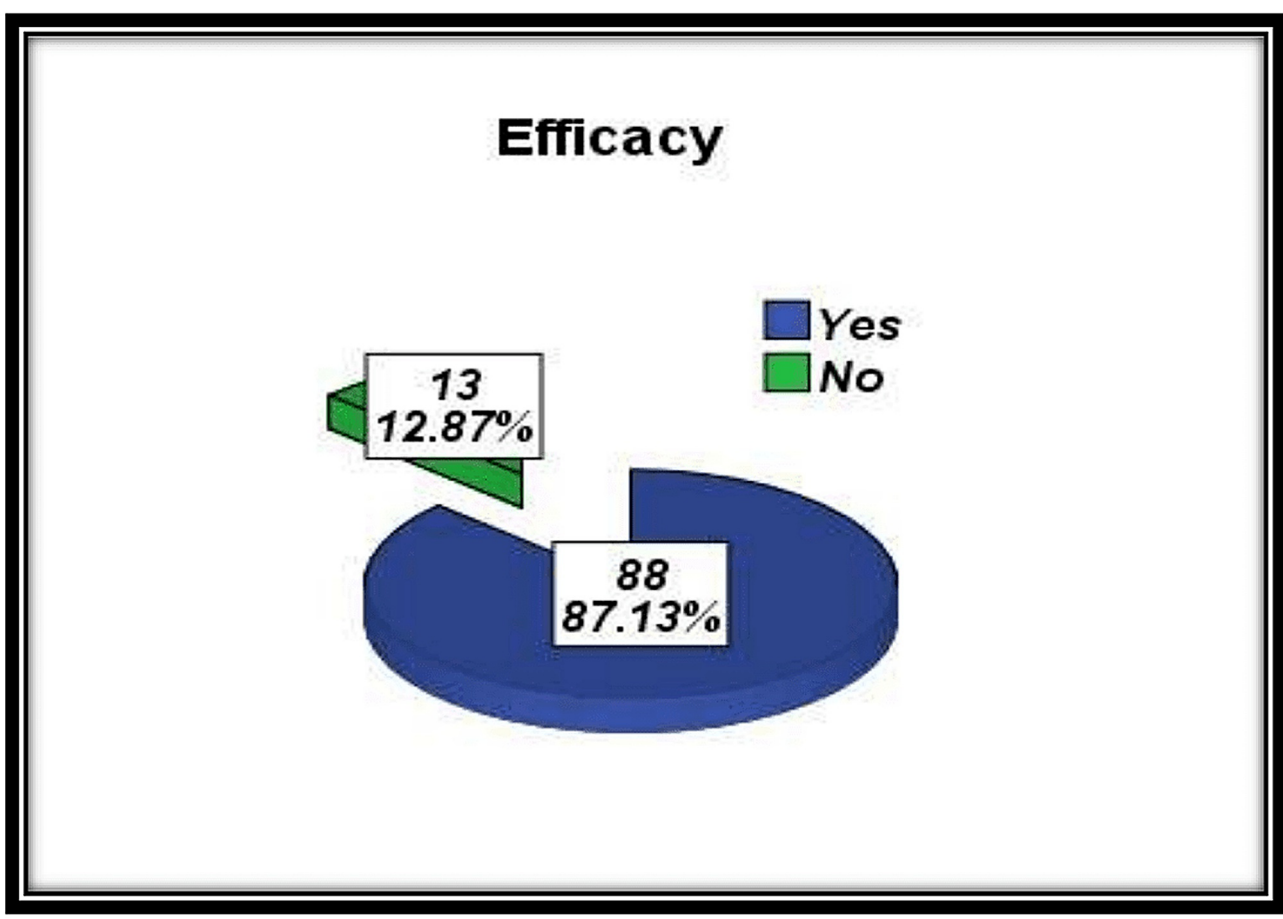
Efficacy of 5-FU used as a primary treatment of pterygium.

Hence efficacy was achieved in 88 (87.13%) patients but not achieved in 13 (12.87%) patients (Figure 1).

The study results showed ≤30 years patients were 28 in which efficacy achieved in 23 cases and it was not achieved in five cases. Similarly >30 years patients were 73 in which efficacy achieved in 65 cases and it was not achieved in eight cases. Statistically insignificant difference found between the efficacy was age, i.e., p-value = 0.354 (Table 3).

**Table 3:** Comparison of efficacy with age.

		Efficacy		Total
		Yes	No	
Age (years)	≤30	23	5	28
	>30	65	8	73
Total		88	13	101

The study results showed the male patients were 52 in which efficacy achieved in 43 cases and it was not achieved in nine cases. Similarly the female patients were 49 in which efficacy achieved in 45 cases and it was not achieved in four cases. Statistically insignificant difference found between the efficacy was gender, i.e., p-value = 0.170 (Table 4).

**Table 4:** Comparison of efficacy with gender.

		Efficacy		Total
		Yes	No	
Gender	Male	43	9	52
	Female	45	4	49
Total		88	13	101

The study results showed the patients with primary pterygium were 54 in which efficacy achieved in 48 cases and it was not achieved in six cases. Similarly the patients with recurrent pterygium were 47 in which efficacy achieved in 40 cases and it was not achieved in seven cases. Statistically insignificant difference found between the efficacy was type of pterygium, i.e., p-value = 0.571 (Table 5).

**Table 5:** Comparison of efficacy with type of pterygium.

		Efficacy		Total
		Yes	No	
Type	Primary	48	6	54
	Recurrent	40	7	47
Total		88	13	101

On six-month post injection review only four patients in intermediate Grade 2 developed a fleshy grade 3 pterygium and total 26 patients underwent surgical excision of which 13 were those who showed no improvement after the injections.

## Discussion

The standard treatment of pterygium in the literature is surgical excision with a conjunctival graft to cover the defect but recurrence remains as the most common complication after excision [8]. Several adjunctive treatments have been employed to reduce recurrences [9-15]. In our study, we used 5-Fluorouracil, a pyrimidine analogue that causes apoptosis of fibroblasts and has anti-scarring properties. Studies are available that prove its efficacy to treat recurrence or as an adjunct but no study is present where it is used as a primary treatment modality. A study by Said et al. presented that the use of weekly intra-lesional 5-FU injections for the treatment of recurrent pterygium is safe and effective in limiting the progression and inducing the regression of recurrent pterygium [1]. The number of injections can be tailored according to clinical need. Another study showed that 93.3% patients showed regression of fibrovascular tissue (thickness and vascularity) and arrest of progression following a dose of 0.1-0.2 ml of 5-FU in recurrent pterygium [8]. A single intra-operative application of mitomycin C (MMC) has been shown to be effective in reducing the recurrence of pterygium [16]. A study by Prabhasawat et al. resulted that the 5-FU was significantly (P = 0.001) more effective in inhibiting the recurrence of pterygium injected weekly for two weeks compared with the control group at follow-up [17]. Kaplan-Meier survival analysis showed that the recurrence-free period of pterygium in the 5-FU group was significantly (P = 0.005) longer than that of the control group. One study by Valezi et al. suggests that intraoperative infiltration of 5-FU is safe for treatment of pterygium and there is no statistically significant difference between the intraoperative and topical of application of 5-FU, however, the high recurrence rate indicated that other studies would be necessary to show the concentration/dose to better prevent it [18]. Ibrahim and Nada demonstrated that the infiltration of 5-FU rather than topical application as an adjuvant to pterygium surgery is easy, time saving, and does not necessitate copious irrigation with saline as with topical application with comparable results and postoperative complications [19]. Maldonado et al. studied the efficacy of a low dose of intra-operative 5-FU [20]. They concluded that this was ineffective in preventing recurrence. This is probably because of inadequate treatment both in terms of dose and duration, suggesting that a single injection is not sufficient. Adverse effects seen with 5-FU are epithelial keratopathy due to inhibition of mitosis of corneal epithelium but is mostly seen after its use in glaucoma filtering surgery [21]. In our study no epithelial toxicity, sclera thinning or conjunctivitis was seen. Injection-related rather than drug-induced events were noted like stinging sensation and subconjunctival hemorrhage at the site that resolved in around two weeks. Limitation of the study was a longer follow-up that is required to see any recurrence. Grade 1 pterygium and those not yet approaching the cornea were excluded, can be taken into consideration and number of injections can be tailored according to the clinical appearance.

## Conclusions

The use of 5-FU is safe and effective for the treatment of pterygium and it can be used as a primary treatment as the results are promising especially in regards to its improvement in the clinical appearance, prevention of recurrence and decreasing the need of surgical excision and excessive use of steroids which are being used both to treat pterygium and after surgical excision. Studies need to be done to see the response in different geographical regions as sun exposure, occupation and region play an important role in the progression of pterygium. Also cases of pterygium which have not yet approached the cornea can be considered in future studies to see how 5-FU halts its progression with tailored number of injections according to the clinical appearance.
